# Supplementary material for: L-Proline Prevents Endoplasmic Reticulum Stress in Microglial Cells Exposed to L-azetidine-2-carboxylic Acid
Source: Molecules. 2023 Jun 16;28(12):4808. doi: 10.3390/molecules28124808 (PMC10302875; doi:10.3390/molecules28124808)
Supplement: Supplementary file 1 [file molecules-28-04808-s001.zip › molecules-2422861-supplementary.pdf]

L-Proline Prevents Endoplasmic Reticulum Stress in Microglial Cells Exposed to L-Azetidine-2-carboxylic Acid

Jordan Allan Piper [1], Nour Al Hammouri [1], Margo Iris Jansen [1], Kenneth J. Rodgers [2,], Giuseppe Musumeci [3], Amolika Dhungana [4], Sahar Masoumeh Ghorbanpour [4], Laura A Bradfield [4] and Alessandro Castorina [1,*]

Supplementary Figures

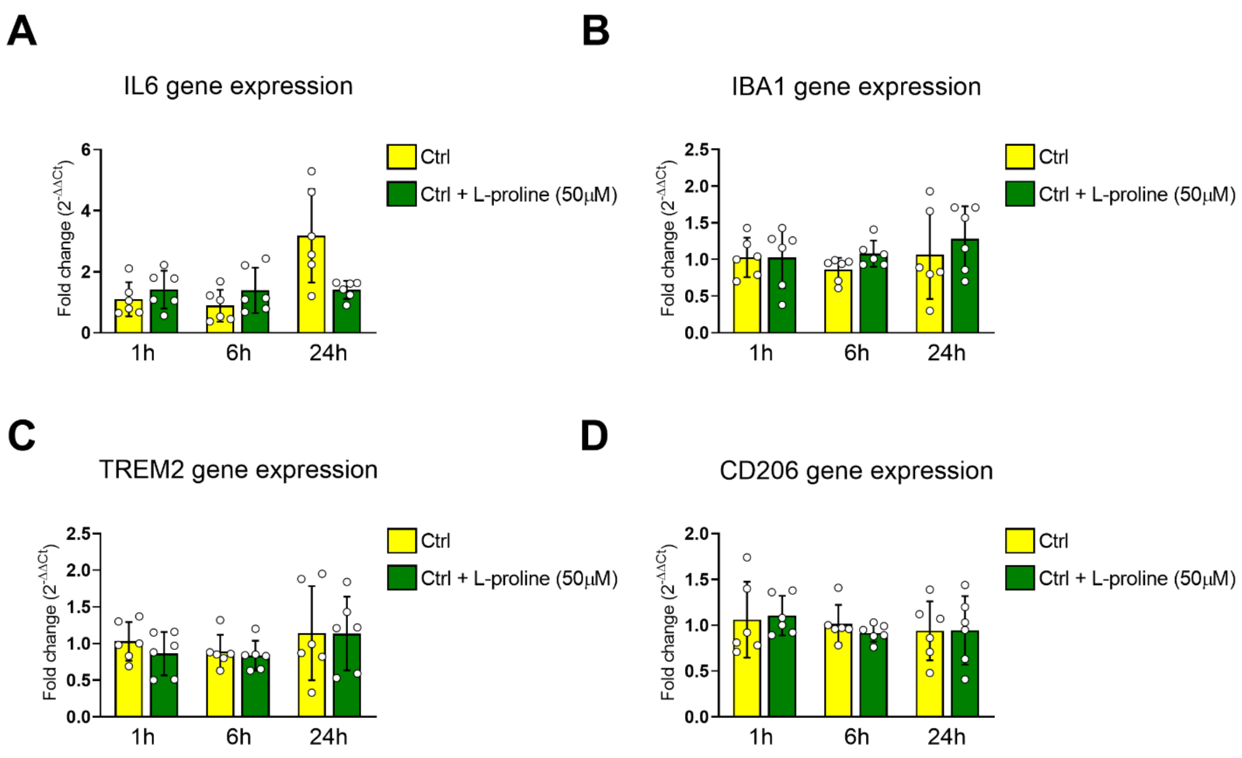

Figure S1. Effects of L-proline treatment alone on the expression pro- and anti-inflammatory genes. Gene expression in untreated cells or treated with L-proline (50 μM) at 1, 6 and 24 h was measured using real-time qPCR and quantified using the ΔCt method after normalization to the S18 housekeeping gene. Bar graphs display comparative gene expression changes of the following inflammation-related genes: (A) IL6, (B) IBA1, (C) TREM2 and (D) CD206. Results are presented as mean fold changes vs Ctrl ± SEM, obtained from (n=6) biological replicates.

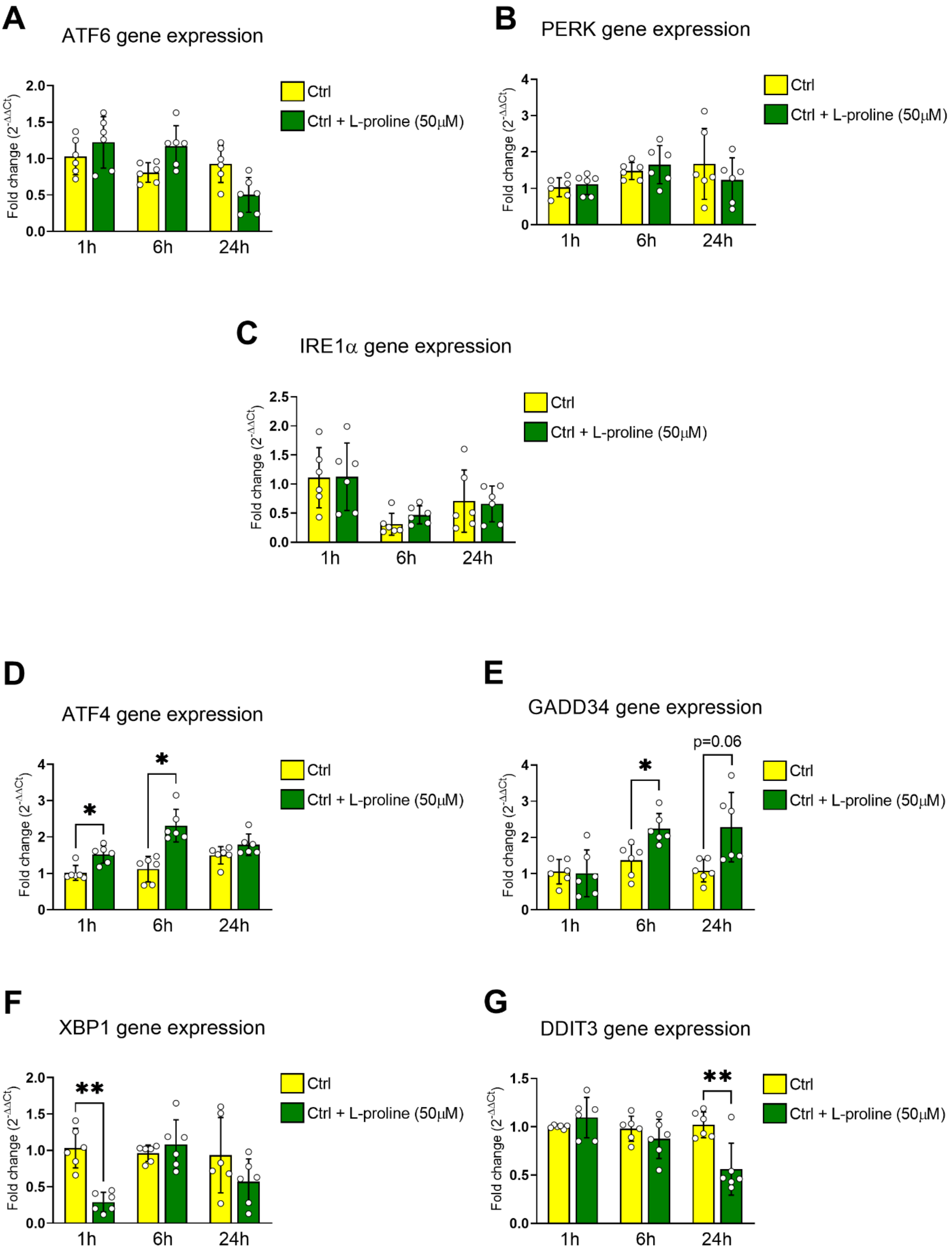

Figure S2. Effects of AZE treatment on the expression of ER stress / UPR genes. Gene expression was measured using real-time qPCR and quantified using the ΔCt method after normalization to the S18 housekeeping gene. Bar graphs show the differential expression of the following upstream and downstream regulators of the ER stress / UPR response; ATF6, PERK (aka EIF2AK3), IRE1α (aka ERN1), ATF4, GADD34, XBP1 and DDIT3. Relative expression levels of (A) ATF6, (B) PERK, (C) IRE1α, (D) ATF4, (E),

GADD34, (F) XBP1 and (G) DDIT3 transcripts were measured in untreated (Control) and L-proline-treated BV2 microglial cells at various times (1, 6 and 24 h, respectively). Results are presented as mean fold changes of Controls ± SEM of three independent experiments, each run using two biological replicates per experiment (n=6). *$p<0.05$ or **$p<0.01$ vs Ctrl, as determined by repeated measures ANOVA followed by Tukey post-hoc tests.
